# The Crossroads between Zinc and Steroidal Implant-Induced Growth of Beef Cattle

**DOI:** 10.3390/ani11071914

**Published:** 2021-06-27

**Authors:** Elizabeth M. Messersmith, Dathan T. Smerchek, Stephanie L. Hansen

**Affiliations:** Department of Animal Science, Iowa State University, Ames, IA 50011, USA; emm2@iastate.edu (E.M.M.); dtsmerch@iastate.edu (D.T.S.)

**Keywords:** cattle, estradiol, growth, hormones, trace minerals, trenbolone acetate, zinc

## Abstract

**Simple Summary:**

This review addresses the physiological and biochemical connections between steroidal implants and Zn and their interaction to influence growth in beef cattle. Steroidal implants have been widely accepted as a growth-promoting technology that provides an unmatched economic return to the producer and improves beef production’s environmental sustainability. Likewise, decades of research have indicated Zn is vital for skeletal muscle growth. Considering Zn is an essential trace mineral, strategic Zn supplementation to implanted cattle may optimize beef production. Although many interrelationships between steroidal implants and Zn are new and forthcoming, the literature reviewed hereafter indicates roles for Zn in a multitude of growth processes pertinent to steroidal implant-induced growth and uncover changes in Zn metabolism due to steroidal implant use.

**Abstract:**

Growth-promoting technologies such as steroidal implants have been utilized in the beef industry for over 60 years and remain an indispensable tool for improving economic returns through consistently improved average daily gain via increased skeletal muscle hypertrophy. Zinc has been implicated in skeletal muscle growth through protein synthesis, satellite cell function, and many other growth processes. Therefore, the objective of this review was to present the available literature linking Zn to steroidal implant-induced protein synthesis and other metabolic processes. Herein, steroidal implants and their mode of action, the biological importance of Zn, and several connections between steroidal implants and Zn related to growth processes are discussed. These include the influence of Zn on hormone receptor signaling, circulating insulin-like growth factor-1 concentrations, glucose metabolism, protein synthesis via mTOR, and satellite cell proliferation and differentiation. Supplemental Zn has also been implicated in improved growth rates of cattle utilizing growth-promoting technologies, and steroidal implants appear to alter liver and circulating Zn concentrations. Therefore, this review provides evidence of the role of Zn in steroidal implant-induced growth yet reveals gaps in the current knowledge base related to optimizing Zn supplementation strategies to best capture growth performance improvements offered through steroidal implants.

## 1. Introduction

Increasing supplementation of trace minerals (TM) [1] and more specifically Zn [2,3,4] to cattle improves gain and hot carcass weight. Trace minerals are important in biology and in particular, Zn is implicated in a vast array of cellular and physiological processes. Zinc acts as a cofactor in numerous enzymes, functions as a component of a wide range of transcription factors, and is involved in nearly every signaling pathway in the mammalian body [5,6]. As more knowledge regarding Zn in the body is elucidated, the breadth and importance of its functions only increase. In malnourished children [7,8] and cattle [9,10], stunted growth has been associated with Zn deficiency. The decrease in growth as a result of malnourishment and Zn deficiency has been linked to the important role Zn plays in protein synthesis, cell proliferation, cell differentiation, and the regulation of DNA synthesis and mitosis [11,12].

Steroidal implants have been used since the 1950s in the U.S. beef industry although they are no longer an accepted growth-promoting technology in several countries. This technology routinely improves cattle gains by 16–20% [13] and is a vital component to improving sustainability in the beef industry. The improvement in growth performance in implanted cattle likely also results in an increase in the Zn required for that animal to accommodate the implant-induced increase in protein synthesis. Furthermore, steroidal implants have been observed to alter both liver and circulating Zn concentrations of steers [1,14], suggesting implants affect Zn metabolism. Therefore, it is imperative to gain a better understanding of how steroidal implants influence Zn metabolism and how supplemental Zn influences steroidal implant-induced growth. The objective of this review is to present the data available to illustrate the relationships and potential interactions between steroidal implants and Zn. Herein the authors discuss the biological functions of steroidal implants and Zn separately before examining the roles of Zn in steroidal implant-induced protein synthesis and cattle growth as well as steroidal implant effects on Zn metabolism.

## 2. Steroidal Implants

### 2.1. Estrogen and TBA in Steroidal Implants

Steroidal implants typically contain a high concentration of steroid compound housed within several small, compressed pellets administered subcutaneously in the back of the ear using an implant needle and applicator. The steroid hormone, once released, enters circulation and is carried to relevant target tissues such as skeletal muscle, adipose tissue, liver, and bone, where it can then elicit a physiological response in the beef animal [15,16]. There is a wide variety of steroidal implant products available containing different active compounds often included in a combination of one another and at varying ratios. These products undergo rigorous testing to ensure the safety of the end product to consumers when used on-label [17]. The majority of steroidal implants contain an estrogenic constituent, either the naturally occurring estradiol-17β (E_2_) or the synthetic prodrug of E_2_, estradiol benzoate (EB). The estrogenic component can be included alone, but most often, it is included in combination with either progesterone, testosterone propionate, or trenbolone acetate (TBA) [16]. The anabolic activity of TBA is 5 to 8 times greater than testosterone; additionally, TBA cannot be aromatized into estrogen in the body like testosterone [18]. The most potent steroidal implants contain TBA alone or in combination with EB or E_2_ and generally contain the highest amount of hormone commercially available [16]. It is well documented that a synergistic relationship between TBA and E_2_ exists which yields greater growth performance responses than TBA or E_2_ alone [19,20].

Many questions remain about the exact mechanisms by which exogenous steroid hormones stimulate a growth response in the body. Previous work suggests growth responses following steroidal implant administration occur via different mechanisms for estrogens and androgens. Classically, the mechanism of action associated with both TBA and E_2_ were described as genomic in nature [21,22]. Recent literature suggests that in addition to the classical genomic steroid hormone response, a much more rapid non-genomic response occurs as well through an unrelated pathway [23,24,25,26].

### 2.2. Genomic Mode of Action

Classically, the androgenic constituent of a steroidal implant is thought to act directly on skeletal muscle via genomic mechanisms through interactions with the androgen receptor (AR) and the insulin-like growth factor-I receptor (IGFR-1). The AR is a ligand-inducible nuclear receptor transcription factor critical in mediating cellular response to androgens by binding hormone regulatory elements of specific genes, ultimately triggering a cascade of transcriptional events [27]. Effectively, this model for steroid hormone action shows steroid hormones cross the plasma membrane to enter the cytoplasm and bind to specific intracellular receptor proteins. The bound steroid receptors act as transcription factors and bind to specific DNA response elements in target gene promoter regions, ultimately resulting in activation or repression of transcription and subsequently protein synthesis [28]. A primary example related to the genomic response as a result of administration of a steroidal implant is the androgen response element located on the promoter region of the insulin-like growth factor 1 (IGF-1) gene in skeletal muscle [22]. Insulin-like growth factor-1 is crucial for stimulating rates of proliferation, differentiation, and protein synthesis as well as decreasing rate of protein degradation in cultured bovine satellite cells.

The classical genomic effect of the estrogenic constituent of steroidal implants is thought to exert its effect on lean tissue accretion in an indirect manner via the somatotropic axis. The genomic mechanism through which estrogens impact skeletal muscle growth involve binding of the receptor-ligand complex to specific response elements in the regulatory region for a particular gene, similar to how androgens affect transcription. The primary way by which E_2_ alters cellular functions is by binding to both estrogen receptor α (ESR1) and β (ESR2) [29,30]. The transcription factor activity of the estrogen response element on the growth hormone-releasing hormone (GHRH) gene in the hypothalamus is of key importance relative to the genomic response elicited by E_2_ in a steroidal implant. Acidophilic cells located in the anterior pituitary gland release growth hormone (GH) in response to GHRH. Estradiol has been shown to increase GHRH release from the hypothalamus and the size of acidophilic cells in the anterior pituitary gland [31,32]. One influence of central importance related to skeletal muscle growth is that E_2_-stimulated release of GH increases hepatic IGF-1 production leading to increased rates of proliferation, differentiation, and protein synthesis as well as whole body growth.

### 2.3. Non-Genomic Mode of Action

In addition to the classical androgen-mediated genomic response a much more rapid, non-genomic response occurs. This non-genomic response is thought to occur via G protein-coupled receptors (GPR). Activation of GPR through testosterone increases both skeletal muscle growth and satellite cell (SC) proliferation in skeletal muscle [33,34]. The activated GPR initiates response of matrix metalloproteinases (MMP), which are rapidly activated through post-translational mechanisms, in turn triggering transcription of genes whose overexpression promotes cell proliferation, hypertrophy and profibrotic processes [35]. The GPR in turn function by way of a secondary messenger system which results in the activation of MMP 2 and 9 that release membrane-bound heparin binding epidermal growth factor-like growth factor (hbEGF). Following release, hbEGF then binds to and activates epidermal growth factor receptors (EGFR) and promotes downstream phosphorylation of protein kinase B (AKT) [25,26]. An increase in the phosphorylation of AKT was observed with TBA treatment of bovine satellite cells (BSC) resulting in improved proliferation and protein synthesis rate [25,26]. This is critical as AKT is a key regulator of protein synthesis via the mammalian target of rapamycin (mTOR) pathway in postnatal bovine skeletal muscle growth. Phosphorylated AKT (pAKT) elicits responses of several downstream signaling molecules including mTOR which, when activated, upregulates protein synthesis. Additionally, pAKT inhibits forkhead box protein (FoxO), preventing downregulation of protein synthesis.

The estrogenic component of a steroidal implant, being a member of the steroid hormone family, also causes a non-genomic growth response in skeletal muscle. The G protein-coupled estrogen receptor-1 (GPER-1) is a member of the GPR superfamily [21,36]. Using a specific GPER1 agonist, G1, Bologa et al. [37] observed IGF-1 mRNA expression in cultured BSC increased. Therefore, GPER-1 is likely to be involved in the E_2_-induced expression of IGF-1 in target cells. The estrogen receptor-ligand complex has been implicated in activating several intracellular pathways, including Raf-1/MAPK kinase (MEK)1/2/ERK1/2 and the PI3K/AKT pathways [29,38]. In a seemingly similar way to how TBA elicits a non-genomic growth response, GPER-1 activates MMP which cleave hbEGF from the cell membrane [39]. Heparin-binding epidermal growth factor-like growth factor binds to surface EGFR to initiate activation of downstream kinases, including ERK1/2 MAPK and AKT [40]. Interestingly, both Zn [41] and Cu [42] have been implicated in perpetuating GPR signaling hinting at further roles for TM to influence GPR and subsequently steroidal implant growth pathways.

## 3. Biological Importance of Zinc in Mammals

### 3.1. Classical Zinc Literature

Zinc functions in mediating whole body growth and protein synthesis. It was demonstrated that in piglets fed Zn deficient diets, growth retardation was evident as early as 1 week following dietary treatment administration [43]. Furthermore, halted growth and a decrease in plasma Zn concentrations have been observed in calves fed diets nearly void of Zn [10]. In a study using weaned male rats, Oberleas and Prasad [44] investigated the relationship between Zn and protein concentrations in feedstuffs. Dietary protein treatments containing 4, 8, 12, 16 or 20% soybean protein were fed and were either supplemented with no additional Zn or 55 mg Zn/kg for a duration of 10 weeks. Of rats given 4 or 8% protein without Zn 75% died, compared with only 8% mortality in the groups given Zn. Adequate Zn concentration allowed for utilization of the increasing amount of protein offered by the dietary treatments. Zinc supplementation effectively allowed for twice the bodyweight gain at protein treatment concentrations of 4, 8, 12 and 16% CP. Additionally, Williams and Chesters [45] found Zn was vital to DNA and protein synthesis. In rats fed a Zn deficient diet for 1 to 5 d, incorporation of [^3^H]thymidine, which functions as a labeled DNA precursor, linearly decreased in the liver, kidney, and spleen, reflective of the importance of Zn in DNA synthesis. Additionally, protein synthesis was markedly decreased in the kidney and spleen of rats, measured by [^14^C]lysine incorporation in these tissues. These studies were instrumental in demonstrating a vital role for Zn in protein utilization in the body.

### 3.2. Zinc Requirement

Official beef cattle TM requirements are established to prevent deficiency and allow for growth [46]. The current Zn requirement for beef cattle is 30 mg Zn/kg on a dry matter (DM) basis [46], however, the work done to establish this requirement occurred over 40 years ago [47]. Interestingly, a survey of consulting nutritionists conducted by Samuelson et al. [48] reports Zn is commonly supplemented at over 300% of NASEM [46] recommendations (100 mg Zn/kg DM). Industry adoption of greater Zn supplementation is practical and low risk due to the low toxicity risk for Zn in cattle as this increase in Zn supplementation is still well below pharmacological supplementation observed in other species [49]. This shift to greater Zn supplementation may be one factor supporting the tremendous improvements in growth rates of the modern beef animal over the last 40 years. Capper [50] provides an extensive review comparing the U.S. beef production in 1977 to beef production in the year 2007. Capper [50] reported cattle in 2007 had a 44% increase in growth rate when compared to cattle from 1977. Since 2007 this trend has not changed; improvements in beef cattle growth rates and carcass weights have continued to increase. Due to the extensive and critical roles Zn plays in relation to proper whole-body growth and protein synthesis it stands to reason that since animal growth rate has improved significantly, so too may the requirement for Zn have also increased in the modern beef animal. However, both growth and dry matter intake (DMI) have consistently increased in beef cattle in the decades since these requirements were established. Meaning that, while recommended dietary TM concentrations have remained largely unchanged, greater total Zn consumption has occurred over this same time period due to increased DMI. For example, Capper [50] reported slaughter body weights of cattle in 1977 to be 468 kg vs. 607 kg for cattle fed in 2007. For this sample calculation slaughter body weight was multiplied by 2.2% to estimate DMI assuming these cattle consumed equal amounts of DM as a proportion of their slaughter body weight the 1977 cattle would have consumed ~10.3 kg DM while 2007 cattle consumed ~13.4 kg DM. Now, assuming these cattle were supplemented Zn at 30 mg/kg DM in accordance with NASEM [46] guidelines, 1977 cattle would have a total daily Zn consumption of 309 mg while 2007 cattle would have consumed 401 mg Zn. Therefore, an increase in mature final body weight results in greater total Zn consumption. However, the question must be posed, does this increase in intake fully address the presumed changes in TM requirements? Do greater concentrations of TM supplementation allow for enhanced growth performance given the large increases in growth performance offered by modern cattle genetics and growth enhancing technologies? To answer these questions and to better determine the TM requirements of cattle based on body composition and growth rate, serial slaughter and TM metabolism studies using modern cattle are necessary. Cattle requirements for Ca and P from NASEM [46] are determined using retained energy and metabolizable protein, this differs from how TM requirements have been determined. Data gained through serial slaughter and TM metabolism studies may allow for similar prediction equations to be developed for TM requirements, thus allowing for more precise TM supplementation. Although this approach may not be applicable to all TM and the current body of literature is limited, the authors believe this is the next step in refining TM requirements in cattle.

### 3.3. Zinc Absorption and Status

Although Zn supplementation is greater across the beef industry [48] than NASEM [46] requirements, absorption of Zn can greatly influence the effects of dietary Zn in cattle. Absorption and movement of Zn throughout the body is reliant upon the ZIP and ZnT family of transporters to move Zn into and out of the cytoplasm, respectively [5]. Zinc is absorbed in the jejunum of the small intestine mostly through apical transporter ZIP4 [51]. Furthermore, the basolateral transporter ZnT1 is responsible for the passage of Zn from the enterocyte into the portal blood [52] where, bound by albumin, it will be transported to the liver to be stored by the protein metallothionein [53]. Dietary supplementation of Zn has been shown to regulate both ZIP4 and ZnT1 expression [51,52]. Therefore, these transporters provide a mechanism for maintaining Zn homeostasis and determine the efficiency of Zn absorption. However, high concentrations of dietary Zn compensate for the down regulation of ZIP and ZnT transporters [54]. Sandström et al. [55] found the fractional absorption of Zn supplemented to humans through radiolabeled Zn in water was decreased with increasing concentrations. Specifically, supplementation of 40 vs. 200 µmol of Zn resulted in absorption rates of 73 or 46%, respectively, though total Zn absorbed increased three-fold for the higher dose. Evidence of dietary Zn altering absorption has also been found in cattle. Steers (309 kg) fed 36 vs. 156 mg dietary Zn/kg DM had greater apparent absorption, though steers fed the greater concentrations of Zn retained over two times as much Zn as steers fed 36 mg Zn/kg DM [56]. However, no differences in Zn apparent absorption or retention were observed with similar Zn treatments in heavier steers (485 kg) [57] indicating Zn absorption may be influenced by stage of growth, though additional research in this area is needed to verify this assumption. These data alongside the basic Zn transporter literature suggest cattle receiving greater concentrations of dietary Zn would have greater total Zn retention than cattle receiving 30 mg Zn/kg DM, irrespective of transporter down regulation.

Yet, Zn absorption may be influenced by additional factors that alter whole body use of Zn, such as growth. Skeletal muscle and bone comprise a large percentage of Zn storage. However, classified as slow Zn metabolizers, release of Zn from muscle and bone is less likely [58]. Seeing that 90% of Zn in the body is thought to reside in tissues with slow Zn metabolism [58], labile Zn stores are limited. Therefore, our interpretation of Zn status based on measurable tissue Zn stores may be unreliable, at best. As such, severe Zn deficiency in calves results in only a small decline in Zn concentrations for some tissues such as liver, bone, and pancreas but no changes in muscle and brain [59]. However, in situations of Zn deficiency, plasma Zn concentrations appear to depict Zn status more accurately. In calves fed a diet nearly void of Zn plasma Zn concentrations dropped, feed intake was depressed, and growth was halted [10]. Furthermore, removal of supplemental Zn to a subset of calves from this same experiment resulted in a sharp decrease in body weight within 2 weeks of Zn removal, even though plasma Zn concentrations started as adequate [10]. These data suggest even cattle starting with adequate plasma Zn concentrations (0.8–1.4 mg/L) [60] can experience detrimental effects of a severely Zn deficient diet. Miller [59] suggests cattle lack a mechanism to quickly mobilize Zn stores, leading to these quick decreases in circulating Zn concentrations. Feeding sub-optimal concentrations of Zn could also result in depletion of Zn status and performance, though effects would be much more difficult to recognize [59]. In modern cattle feeding, severe Zn deficiencies are unlikely due to ample Zn supplementation [48], unless antagonists (Ca, Cu, or Fe) are readily present in the diet [46,49]. Therefore, circulating Zn concentrations for cattle across the industry are likely adequate but management strategies altering growth rates such as steroidal implants may result in short term inadequate circulating Zn concentrations due to increased Zn utilization in protein synthesis to accommodate increased growth. This marked increase in Zn demand could result in depleted circulating Zn concentrations as other Zn stores are not as labile. Therefore, steroidal implants could influence Zn absorption as low plasma Zn concentrations would eventually trigger greater uptake of Zn by the enterocytes to support the increased Zn demand.

## 4. Interconnections between Steroidal Implants and Zinc

### 4.1. Hormone Receptors and Downstream Signaling

Zinc has been implicated in many pathways downstream of hormone receptors often with a role in affecting the phosphorylation state of the cell, these responses typically begin at the receptor. Similar to the non-genomic pathways by which hormone signaling occurs, Zn treatment can also directly influence GPER signaling. Interestingly, Pisano et al. [61] found the inhibition of GPER halted the Zn-induced phosphorylation of EGFR and IGF-1R as well as the downstream activation of ERK and AKT. Therefore, these data indicate Zn induces ERK and AKT activation through GPER signaling. In human breast cancer cells, Zn was shown to phosphorylate EGFR and IGF-1R leading to the downstream activation of ERK and AKT in a dose-dependent manner [61]. These data agree with previous observations of increased EGFR phosphorylation due to Zn supplementation [62,63,64]. However, the exact mechanism by which Zn influences GPER and its downstream signaling is not known. Pisano et al. [61] suggests Zn stimulation of GPER is mediated through Zn-induced reactive oxygen species production, but evidence of this relationship requires further examination. Though it is clear Zn treatment influences the E_2_ receptor’s signaling in some manner.

Treatment of normal human bronchial epithelial cells with 100 µM Zn^2+^ for 2 h resulted in increased release of hbEGF from EGFR, resulting in EGFR phosphorylation [65]. Phosphorylation of specific tyrosine kinase residues is vital to EGFR downstream signaling by promoting binding of the receptor by proteins that lead to cell proliferation and migration, among other aspects of growth [66]. Zinc supplementation increases phosphorylation of EGFR at the autophosphorylation sites Tyr1068 and Tyr1173 as well as Tyr845, a known transphosphorylation site [64]. Furthermore, utilizing an MMP inhibitor to test the mechanism of hbEGF release, Wu et al. [65] observed a decrease in Zn-induced but not EGF-induced EGFR phosphorylation. This suggests Zn-induced phosphorylation of EGFR is mediated through MMP. Interestingly, mRNA gene expression of MMP increases with greater Zn treatment [67,68]. As a family of Zn-dependent enzymes [69,70], MMP are known for cleaving membrane-bound hbEGF [71,72]. Further investigation implicated MMP3 as the responsible enzyme due to an association between the release of hbEGF from EGFR and an increase in free MMP3 in normal human bronchial epithelial cells treated with Zn [65]. Although MMP2/9 have already been linked to cleaving membrane-bound hbEGF [25,26], these data suggest MMP3 is involved in Zn-induced phosphorylation of EGFR through release of hbEGF from its binding site on EGFR. However, validation of these results within muscle cells treated with steroidal hormones is necessary to link Zn-induced MMP3 actions to implant-induced growth further.

Although Zn has not been directly related to androgen receptor function like the previously discussed estrogen receptor, a role for Zn in androgen signaling is emerging. Interestingly, the Zn transporter ZIP9 was discovered as a membrane androgen receptor that facilitates non-genomic signaling in fish [73] and human breast and prostate cancer cells [74]. The direct mechanism of action is not clear. However, ZIP9 has been observed to activate G proteins and increase intracellular concentrations of cAMP, a GPR activated intracellular signaling molecule, when cells were treated with testosterone [73,75]. Additionally, testosterone binding appears to activate the Zn transporter function of ZIP9 as intracellular concentrations of Zn were increased in cultured granulosa and theca cells treated with testosterone [73]. Knocking ZIP9 out provided additional evidence of testosterone’s effects on ZIP9 function, as Zn transport was mitigated by the knockout [73,75]. However, Converse and Thomas [75] found intracellular Zn concentrations and incidence of apoptosis mediated by ZIP9 were dependent upon the stage of growth in granulosa and theca cells. Mediated through testosterone binding of ZIP9, early-stage follicles exhibited lesser concentrations of intracellular Zn and cAMP and less apoptosis than late-stage follicles [75]. These data suggest Zn may play a dynamic role in cellular signaling dependent upon stage of growth. The discovery of ZIP9 as not only a transporter of Zn but as an androgen receptor makes this protein an intriguing target for future studies examining the interaction between Zn and steroidal implants. It is apparent Zn influences both E_2_ and androgen hormone receptor signaling.

### 4.2. Insulin-Like Growth Factor-1

Steroidal implants have consistently been shown to increase both hepatic and/or local skeletal muscle IGF-I production in steers implanted with a combination TBA + E_2_ implant [76,77,78]. Insulin-like growth factor 1, through the PI3K/AKT/mTOR and PI3K/AKT/GSK3β pathways, has been shown to be a potent stimulator of protein synthesis in skeletal muscle and, at the same time, can decrease the rate of protein degradation [79]. Additionally, local IGF-I is important for recruiting bovine satellite cells to supply additional nuclei necessary to support postnatal skeletal muscle hypertrophy [15]. The importance of locally produced IGF-1 was examined by Liu et al. [80], who reported that, in mice, knockout of the liver-specific IGF-1 gene resulted in lesser concentrations of circulating IGF-I. However, it is of interest that these IGF-1 knockout mice sustained normal postnatal growth thus signifying the integral role local IGF-1 plays in skeletal muscle.

Although Zn supplementation has been shown to improve growth rates in rats [44], links between Zn and growth pathways in the body are still not fully understood. Considering the GH-IGF system is well known for its regulation of growth [81], it seems likely Zn may influence IGF-1 signaling. Much of the work detailing the relationship between Zn and IGF-1 has taken place in Zn deficient children and rodents. Dorup et al. [82] investigated the role of IGF-1 and GH in growth inhibition using 3-week-old rats fed a Zn-deficient diet for 14 d. The Zn-deficient diet caused an 83% drop in weight gain compared with pair-fed controls and decreased serum Zn and serum IGF-1 by 80 and 69%, respectively.

The majority of circulating IGF-1 is bound by a binding protein that acts to prolong the half-life of IGF-1 [83]. Due to the many roles of IGF-1, binding proteins guard against unwarranted IGF-1 stimulation [84]. Therefore, measurements of circulating IGF-1 are likely correlated with circulating IGFBPs such as IGFBP3 [83]. Both Cesur et al. [85] and Hamza et al. [8] observed serum IGF-1 and IGFBP-3 below the normal reference range in over 94% of Zn deficient children considered short for their age group. However, rats fed very low (<1 µg Zn/g) or low concentrations of Zn (7 µg Zn/g) had no differences in serum IGF-1 or IGFBP-3 vs. control fed rats (25 µg Zn/g) [86]. Furthermore, supplementation of Zn increased the height and weight of previously Zn deficient children [7], while Hamza et al. [8] only observed an improvement in height of Zn supplemented children. Differences may have been attributed to the length of Zn supplementation as Ninh et al. [7] supplemented for 5 mo compared to only 3 mo by Hamza et al. [8]. Interestingly, Hamza et al. [8] fed nearly 5 times more Zn per day to children than Ninh et al. [7].

Circulating concentrations of IGF-1 and/or IGFBP-3 have been observed to increase with Zn supplementation [7,8,87,88]. Rats fed low concentrations of Zn (7 µg Zn/g) had greater liver IGF-1 and IGFBP-3 mRNA gene expression than rats fed the control diet containing 25 µg Zn/g [86]. Though, serum glucose concentrations were greater for rats fed < 1 µg Zn/g than rats pair-fed the control diet (25 µg Zn/g) [86]. These data indicate Zn status of the animal influences IGF-1 concentrations and suggests a relationship between Zn and glucose metabolism. It is important to note much of this work was conducted in severely Zn deficient models, and though these data contribute to the understanding of the mechanisms by which Zn influences IGF-1, these severe deficiencies are unlikely to be observed in beef cattle populations. Therefore, this presents a challenge with regard to applying these data to beef cattle production; further work is warranted to determine if dietary Zn supplementation provides an additive effect on circulating IGF-1 concentrations alongside the effects of implants. Furthermore, these data indicate body Zn concentrations and supplemental Zn impact IGF-1 concentrations. Alongside increases in IGF-1 concentrations due to steroidal implants, Zn may synergistically increase IGF-1 concentrations and subsequently stimulate growth processes.

### 4.3. Glucose Metabolism in Muscle

As previously suggested, the literature strongly indicates a role for Zn concerning glucose metabolism. While ruminants derive a substantial amount of energy from volatile fatty acids, gluconeogenesis plays an important role in ruminant energy metabolism [89]. Glucose metabolism at the level of skeletal muscle is of interest as it pertains to livestock production as glucose is an important energy source to cells throughout the body, including skeletal muscle tissue, which is the primary site of insulin-stimulated glucose uptake in the fed animal [89,90]. Effective glucose homeostasis in the body is a product of the hormone insulin, glucagon, and the insulin sensitivity of a particular target tissue [91]. It is known that steroidal implants elicit an effect on the rate of protein synthesis and degradation as well as a profound effect on the extent of skeletal muscle hypertrophy [19]. With increased protein synthesis and greater accumulation of lean tissue, a concurrent increase in energy demand occurs to sustain this degree of hypertrophy. Therefore, it is of interest to this review to further investigate and detail the role Zn plays in insulin production and glucose homeostasis in the body and how this impacts growth performance responses elicited by steroidal implants.

The behavior of proinsulin and insulin in the presence of Zn suggests Zn is important in β-cell production of insulin for most animal species. Zinc is an essential TM that plays a vital role in the formation of insulin, stimulation of phosphorylation of the β-subunit of this hormone receptor, activation of PI3K, and the translocation of glucose transporter 4 (GLUT4) [92,93]. The transporter GLUT4, which is present in muscle and adipose tissue, is insulin-sensitive and facilitates postprandial blood glucose uptake into muscle and adipose tissues [90]. Tang et al. [94], using 3T3-L1 adipocyte cells, showed that Zn treatment of cells caused increased tyrosine phosphorylation of the insulin receptor β subunit and phosphorylation of AKT, and this was concomitant with enhanced glucose transport, independent of insulin. This observation is further supported by Miranda and Dey [95], who showed that in C2C12 skeletal muscle cells, Zn elicited an insulin mimetic response by phosphorylating tyrosine and the insulin receptor substrate 1 (ISR1) without insulin present.

Wu et al. [96] showed that Zn effectively stimulated glucose uptake in both normal as well as insulin-resistant myotubes, and this occurred along with the upregulation of AKT, the translocation of GLUT4, and the phosphorylation of Gsk3β which is a substrate in the AKT pathway that helps mediate glucose metabolism. A study was conducted where gene expression of ZIP7, a primary intracellular modulator of Zn homeostasis that increases the cytosolic Zn concentration by mobilizing Zn from the extracellular space or intracellular stores, was knocked down in C2C12 myoblasts. Concomitant with ZIP7 downregulation, mRNA expression for many genes involved in glucose metabolism, including GLUT4, were lessened [97]. In skeletal muscle, GLUT4 plays an important role in transporting glucose across the plasma membrane, where it can be utilized as an energy source via glycolysis or stored as glycogen, though glycogen storage is lesser in ruminants compared to monogastric species [98,99]. Thus, decreased GLUT4 protein observed in C2C12 cells where ZIP7 had been downregulated indicates a decrease in glucose transport and subsequent genes associated with both glycolysis and glycogenesis pathways. Therefore, this indicates an important role for Zn transport related to glucose metabolism.

Additionally, Ohashi et al. [100] reported Zn and insulin function synergistically to enhance satellite cell activation and phosphorylation of AKT. When evaluated in vitro using C2C12 cells and treating with Zn and/or insulin it was observed that pAKT was increased and sustained compared to treatment with either Zn or insulin alone, suggesting a synergistic relationship. Although innate differences exist between ruminant and monogastric glucose metabolism, these data indicate Zn plays an important role in insulin production, glucose uptake, and AKT phosphorylation. The strong body of literature linking Zn to energy metabolism is of importance as one considers how Zn may impact the growth performance responses in cattle elicited by steroidal implants.

### 4.4. Protein Synthesis Pathways

**Figure 1:**
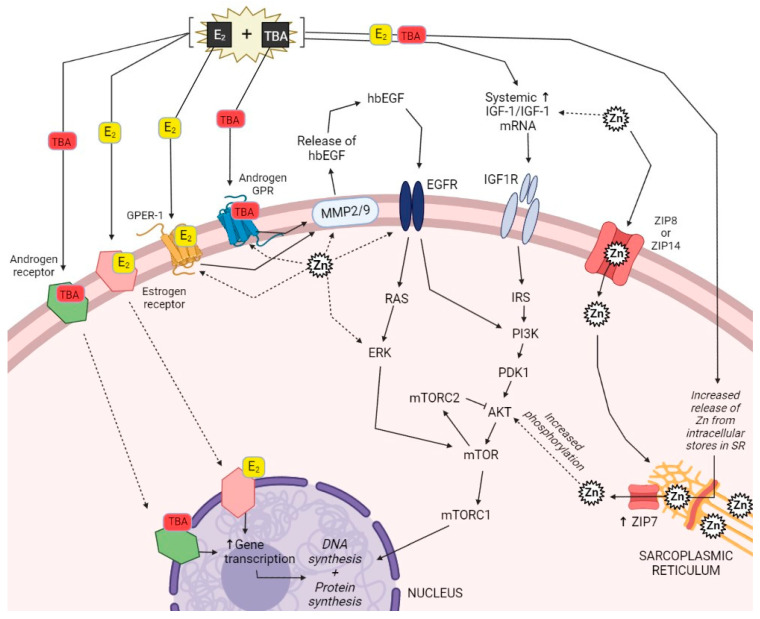
Proposed physiological pathway interactions of combination E_2_ + TBA steroidal implants and Zn in skeletal muscle cells as discussed in this review. Specific focus is placed on the genomic and non-genomic steroid hormone pathways, protein and DNA synthesis pathway, and the corresponding points at which evidence suggests Zn interacts with these pathways. Definitions: AKT (Protein kinase B), Androgen GPR (Androgen specific G protein-coupled receptor), E_2_ (Estradiol), EGFR (Epidermal growth factor receptor), ERK (Extracellular signal-regulated kinase), GPER-1 (G protein-coupled estrogen receptor 1), hbEGF (Heparin binding epidermal growth factor-like growth factor), IGF-1 (Insulin-like growth factor 1), IGF1R (Insulin-like growth factor 1 receptor), IRS (Insulin receptor substrate), MMP2/9 (Matrix metalloproteinase 2/9), mTOR (Mammalian target of rapamycin), mTORC1 (Mammalian target of rapamycin 1), mTORC2 (Mammalian target of rapamycin 2), PDK1 (Phosphoinositide-dependent kinase-1), PI3K (Phosphoinositide 3-kinases), RAS (Ras family of related GTPase proteins), SR (Sarcoplasmic reticulum), TBA (Trenbolone acetate), ZIP7 (Zinc transporter SLC39a7), ZIP8 (Zinc transporter SLC39a8), ZIP14 (Zinc transport SLC39a14).

Downstream of GH and IGF-1 signaling, a considerable body of the literature reveals additional interactions for Zn and steroidal implants in protein synthesis exist (Figure 1). Steroid hormones in implants have been well documented in their ability to increase protein synthesis and reduce protein degradation, thus improving net protein synthesis [101,102,103] via hormone receptors to stimulate protein synthesis pathways such as IGF-1, IGFR-1, PI3K, AKT, and mTOR. Phosphorylated AKT increases protein synthesis by activating the mammalian target of rapamycin (mTOR) pathway. The mTOR cascade plays a primary regulatory role related to cell growth, cell cycle progression, and protein synthesis [104]. While pAKT increases the rate of protein synthesis, it also inhibits protein degradation by repressing transcription factors of the FoxO family which have an inhibitory effect on the mTOR pathway [105]. Thornton et al. [26] observed that BSC cultures treated with TBA, hbEGF, or LR3-IGF-1 had greater pAKT protein abundance when compared to control cultures that did not receive a treatment. Additionally, treatment of fused BSC cultures with E_2_ resulted in a dose-dependent increase in protein synthesis rate coupled with a decrease in protein degradation rate [106].

Zinc appears to be relevant to many of these same protein synthesis pathways. Nimmanon et al. [107] reported a role for ZIP7 in supporting cell survival and proliferation. Phosphorylation of ZIP7 results in Zn release from intracellular stores, which activates multiple tyrosine kinases as well as ERK1/2 and AKT. Nimmanon et al. [107] showed a Zn treatment-induced increase in EGFR and erbB2 tyrosine phosphorylation, providing yet another example of Zn increasing the phosphorylation state of the cell. Therefore, the authors used this evidence to propose the ZIP7-mediated release of Zn from intracellular stores activates multiple signaling pathways involved in promoting growth and proliferation. When an extracellular stimulus activates a cell, Zn is released from intracellular stores, such as the sarcoplasmic reticulum (SR), resulting in activation of different tyrosine kinase pathways through the inhibitory action of Zn on protein tyrosine phosphatases. It has been established that ZIP7 plays a role in the activation of the P13K/AKT pathway [107], whereby impairment of ZIP7 hindered P13K/AKT activation resulting in fewer multinucleated myofibers, and decreased myotube development [108]. This suggests mobilization of Zn from the SR may be necessary for proper muscle hypertrophy. The crossroads between Zn and steroidal implants related to protein synthesis certainly require investigation to clarify the complex inner workings of this synergistic relationship. It is likely the resulting skeletal muscle hypertrophy that follows steroidal implant administration is a sufficient external stimulus to increase Zn demand, triggering Zn release from intracellular stores. These data indicate Zn plays a crucial role in the hormone-induced PI3K/AKT/mTOR protein synthesis pathway. Further elucidation of this relationship in beef cattle is of chief importance as optimizing growth potential of the animal continues to be at the forefront of precision cattle feeding.

### 4.5. Satellite Cell Proliferation and Differentiation

As it pertains to cattle, BSC provide the additional nuclei source necessary to support postnatal skeletal muscle fiber hypertrophy and are critical in determining the extent of muscle growth [30]. Satellite cells can proliferate in response to injury, differentiate, fuse to provide new nuclei, support new myofiber formation, and give rise to regenerated muscle as well as to more satellite cells [109]. Treatment of BSC with TBA increases proliferation [25,26,110,111], while testosterone increases both proliferation and differentiation in C2C12 skeletal muscle myoblasts from mice [112]. Estradiol treatment increases proliferation rate in fused BSC [39] while suppressing myogenic differentiation in C2C12 skeletal muscle myoblasts from mice [113].

In addition to playing a structural role, Zn appears to have an important regulatory function related to cell proliferation and differentiation in satellite cells that likely interacts or overlaps with the mechanisms by which TBA and E_2_ influence satellite cell function. While it is clear Zn plays a role in satellite cell function, past evidence provides controversy about the exact relationship of Zn to proliferation and differentiation of myoblasts. Differential expression of Zn transporters were analyzed by Paskavitz et al. [114] to determine how Zn is distributed during skeletal muscle differentiation. Intracellular concentrations of Zn fluctuate and are tightly controlled by the different ZnT and ZIP transporters in the cell. Paskavitz et al. [114] demonstrated that whole-cell Zn content in C2C12 myoblasts decreased when the differentiation program was initiated. However, after 12 h of differentiation, a gradual increase in cytosolic Zn was detected, and by 72 h a maximum concentration was reached. The gradual and sustained increase in cytosolic Zn^2+^ indicates cellular Zn concentration is dynamic during the myogenesis process. Petrie et al. [115] found insufficient Zn in cultured myoblasts can impair myogenic differentiation.

Additionally, Petrie et al. [116] reported there is likely a potential role for Zn in the initiation of the differentiation process rather than modifying overall mRNA concentrations. Mnatsakanyan et al. [108], using murine myoblasts (C2C12), investigated the effects of exogenous Zn in the form of ZnCl_2_ on myogenic proliferation and differentiation and found that exogenous Zn treatment increased both myoblast proliferation and differentiation. Mnatsakanyan et al. [108] also reported that intracellular Zn concentrations increase in differentiated myoblasts. Ohashi et al. [100] found Zn treatment enhances proliferation in C2C12 myoblasts. However, contrary to some previously reported findings, Ohashi et al. [100] reported treating C2C12 myoblasts cultured in a Zn-free growth medium with exogenous Zn suppressed myogenic differentiation of C2C12 myoblasts. While the effect of Zn on the mitogenic activity regarding cell proliferation was similar between Mnatsakanyan et al. [108] and Ohashi et al. [100], contrasting results were reported with regard to myoblast differentiation. Additionally, Hergenreder et al. [117] conducted a trial where steers were treated with 720 mg·steer^−1^·d^−1^ of Zn as Zn-methionine. Skeletal muscle SC, extracted and cultured from these steers, possessed increased Pax7 gene expression. This indicates animals treated with Zn-methionine had greater capacity to increase total nuclei present in skeletal muscle through proliferation and differentiation of the Pax7 SC. Although some of the previous literature is in disagreement with regard to how Zn specifically impacts different aspects of SC lineage, these data do indicate that Zn does in fact play a role in SC proliferation and differentiation and require further investigation.

### 4.6. Cattle Growth

Although TM recommendations for beef cattle were recently updated [46], few changes have been made to requirements over the past 40 years [47]. However, supplementation of TM observed across the beef industry are commonly 2 to 3 times the NASEM [46] recommendations [48]. Niedermayer et al. [1] supplemented Zn, Cu, Mn, Se, and Co from inorganic sources at 2 to 3 times NASEM [46] recommendations resulting in improved gain and hot carcass weight over non-supplemented and NASEM supplemented steers regardless of implant administration. In this study, due to feeding elevated concentrations of multiple TM simultaneously, it is difficult to determine the growth performance enhancing effects of individual TM, however, increasing TM supplementation did improve growth rates of feedlot cattle. While the impact of Zn on growth performance in this particular study cannot be pinpointed, the role Zn plays in growth, and numerous other physiological and biochemical pathways, made it a suitable candidate for follow up studies. Table 1 outlines several studies detailing the effects of steroidal implants on growth performance as well as Zn metabolism.

Indeed, Zn supplementation influences steroidal implant-induced performance of cattle. When 200 mg Zn/kg DM from ZnSO_4_ was fed to heifers and steers, steroidal implant administration improved average daily gain (ADG) by more than 17% [118]. However, Zn source may influence this response as supplementation of 200 mg Zn/kg DM from Zn-methionine resulted in 26% lesser gains for implanted heifers than non-implanted heifers and no differences in ADG between implanted and non-implanted steers [118]. Interestingly, supplementation of an amino acid chelated Zn blended with ZnSO_4_ (100 mg Zn/kg DM) resulted in a 24% improvement in ADG over non-implanted steers fed the same Zn treatment [119]. Both of the aforementioned studies fed high grain diets typical for finishing phase beef feedlot cattle in the U.S. It is not apparent why Zn source would result in such drastic differences in implant-induced performance between studies as well as between steers and heifers when diet type and days on feed were similar between studies. Follow-up work to answer these questions has not been conducted to the authors’ knowledge. Interestingly, supplementation of up to 150 mg Zn/kg DM from ZnSO_4_ resulted in a linear improvement in performance of steers administered a high potency implant during the first 18 d post-implant administration. In contrast, no differences in performance were observed due to Zn supplementation within non-implanted steers [120]. These data mirror those observed by Genther-Schroeder et al. [4] in which steers receiving a beta agonist linearly improved performance due to increasing Zn supplementation up to 180 mg Zn/kg DM while no performance response to Zn supplementation was observed within cattle not receiving the beta agonist. Collectively, these two studies suggest cattle experiencing high growth rates may respond to additional Zn supplementation indicating a greater demand for Zn. Therefore, strategic supplementation of Zn in combination with steroidal implants may function synergistically to improve growth of feedlot cattle.

Although supplementation of Zn to cattle utilizing growth-promoting technologies has been proven advantageous, timing of Zn supplementation may be vital to capturing this growth response. Peak payout of hormone from steroidal implants occurs within 40 d of implant administration [19], resulting in a period with the greatest growth potential for the implanted animal. The improved performance response observed within 18 d of implant administration by Messersmith and Hansen [120] is likely a result of peak hormonal payout as the performance response disappears over time. Furthermore, effects of hormonal payout were observed in heifers implanted with an extended-release implant on d 0 or a high potency implant on d 0 and again on d 91. During peak hormone payout heifer performance was improved due to increased Zn supplementation from ZnSO_4_ (30 vs. 100 mg Zn/kg DM) [121]. These data suggest strategic supplementation of increased Zn during periods of peak hormonal payout or during other times of high growth rates may improve growth performance. However, additional research is necessary to elucidate how increasing supplemental Zn supports high growth rates and the optimal timing of increased Zn supplementation.

### 4.7. Zinc Metabolism

Although effects of Zn supplementation on cattle performance clearly indicate the importance of Zn in growth, changes in liver and plasma Zn concentrations provide compelling evidence of modified physiological Zn requirements due to steroidal implants. In lambs implanted with zeranol (12 mg), Zn retention was increased by 60% [122]. Furthermore, a positive correlation between Zn and N retention in feedlot steers [57] corroborates a connection between growth and Zn requirements.

Recent studies have found increasing evidence of implants altering Zn requirements by assessing plasma and liver Zn concentrations (Table 1). It is important to note that dietary Zn concentrations for each of the following studies [14,120,123] were above NASEM (30 mg Zn/kg DM) [46] recommendations (40 to 137 mg Zn/kg DM) indicating the effect on plasma Zn associated with implant was minimally influenced by dietary Zn supplementation. Messersmith [14] found implanted steers decreased plasma Zn concentrations by 11.2% on d 13 post-administration of a high potency steroidal implant coinciding with a 29% greater ADG in implanted steers during the first 14 d post-implant. Furthermore, plasma Zn concentrations of implanted steers remained lesser than non-implanted steers through d 73 of the study [14]. These data agree with the 6% drop in plasma Zn and 9.6% increase in ADG observed by Messersmith and Hansen [120] on d 18 when the same high potency terminal implant was utilized. Similarly, heifers receiving a second high potency steroidal implant had 5% lesser plasma Zn 14 d post-implant administration [123]. Interestingly, when plasma Zn concentrations were evaluated throughout a study using a moderate potency implant followed by a high potency implant, plasma Zn concentrations were only lesser for implanted steers 14 d post-administration of the high potency implant [119] suggesting a potency-dependent effect of steroidal implants on plasma Zn concentrations.

**Table 1:** Review of literature examining steroidal implants and zinc metabolism in cattle.

Ref.	Sex	Steroidal Implant ^1^	Potency ^2^	Supplemental Zn ^3^	Day ^4^	∆ ^5^ ADG, %	∆ ^5^ Plasma Zn, %	∆ ^5^ Liver Zn, %
[1]	Steer	200 mg TBA + 20 mg E_2_	High	0	69	--	--	+10.6
				30 ^a^	69	--	--	+11.0
				100 ^a^	69	--	--	+22.9
[14]	Steer	200 mg TBA + 20 mg E_2_	High	30 ^a^	13/14	+29.0	−11.2	−6.6
[118]	Steer	200 mg P + 20 mg EB	Moderate	0	59	+7.6	+1.8	−26.1
				200 ^a^	59	+17.4	+3.2	+30.7
				200 ^b^	59	--	−15.0	+8.5
[118]	Heifer	200 mg TP + 20 mg EB	Moderate	0	50	+11.0	+7.0	−38.2
				200 ^a^	50	+17.0	−2.5	+25.4
				200 ^b^	50	−26.0	+24.7	−40.8
[119]	Steer	80 mg TBA +16 mg E_2_ (Initial)	Moderate	100 ^c^	14/15	--	−3.1	−1.0
		200 mg TBA + 20 mg E_2_	High	100 ^c^	14/15	--	−12.8	+4.8
[120]	Steer	200 mg TBA + 20 mg E_2_	High	0	18	+8.0	−4.6	--
				30 ^a^	18	+3.1	−9.7	--
				100 ^a^	18	+4.5	−7.0	--
				150 ^a^	18	+21.6	−3.5	--
[124]	Steer	200 mg TBA	High	55 ^c^	2	--	−4.3	−16.2
		120 mg TBA +24 mg E_2_	Moderate	55 ^c^	2	--	−8.5	−16.2
[125]	Steer	200 mg P + 20 mg EB (Initial)	Moderate	360 ^d^ mg·steer^−1^·d^−1^	28	--	−4.8	+12.2
		80 mg TBA +16 mg E_2_	Moderate	360 ^d^ mg·steer^−1^·d^−1^	56	--	−7.7	+4.7

^1^ Steroidal implant hormone concentrations are listed using the following abbreviations: E_2_: estradiol, EB: estradiol benzoate, P: progesterone, TBA: trenbolone acetate, TP: testosterone propionate. All steroidal implants were given as the terminal implant, unless otherwise noted as initial implant. ^2^ Steroidal implant potency is arbitrarily categorized as low, moderate, or high based on hormone combination and dose. ^3^ Supplemental Zn is expressed as mg/kg DM, unless otherwise stated. Source of Zn supplemented is represented by superscript: ^a^ inorganic, ^b^ organic, ^c^ blend of inorganic and organic, ^d^ means of inorganic and organic Zn supplementation were combined due to no source differences. ^4^ Represents day relative to administration of the steroidal implant used in each study. ^5^ The percent delta (∆) represents the effect of steroidal implant compared to non-implanted cohorts on growth and Zn metabolism parameters on the noted day post-steroidal implant administration. Changes in plasma Zn concentrations for [118] represent changes in serum Zn concentrations.

In contrast, Huerta et al. [118] observed an increase in serum Zn concentrations throughout the 120 d study in heifers implanted with two moderate potency implants. However, 14 d post-administration of the second implant, serum Zn concentrations for heifers supplemented 0 or 200 mg Zn/kg DM from Zn-methionine appeared to decrease due to implant administration [118]. Yet, these data differ from steers administered a similar steroidal implant and fed the same dietary Zn treatments. In the companion study utilizing steers, Huerta et al. [118] observed no differences in serum Zn due to implant administration in steers. Sex differences may have driven these different responses. Utilization of intact heifers, regardless of melengestrol acetate supplementation to suppress estrus, presents many challenges when assessing the effects of steroidal implants. Mainly, endogenous E_2_ cannot be quantified and accounted for in a controlled study. Therefore, the effects of steroidal implants on Zn metabolism in heifers may not be as reliable as in steers or spayed heifers.

In addition to circulating Zn concentrations, steroidal implants have been found to influence concentrations of Zn in the liver. As previously mentioned, liver Zn concentrations are more difficult to change than plasma due to the less labile nature of Zn stored in the liver. Interestingly, Messersmith et al. [124] observed liver Zn concentrations in steers were decreased within 2 d of steroidal implant administration indicating steroidal implants rapidly change Zn metabolism. In conjunction with the plasma Zn response reported earlier, Messersmith [14] observed a decrease in liver Zn concentrations 14 d post-administration of a high potency implant, though this effect was not evident by d 62. Likewise, Messersmith and Hansen [120] found no differences in liver Zn concentrations 55 d post-implant administration. However, steers administered two moderate potency implants had greater liver Zn concentrations 59 and 74 d post-implant administration when supplemented 200 mg Zn/kg DM from ZnSO_4_ or Zn-methionine [118]. In comparison, steers fed no supplemental Zn had decreased liver Zn concentrations due to implant at the same time points [118]. This response may have been driven by the bulk supplementation of Zn increasing Zn storage. However, it is interesting that non-supplemented steers observed an opposite response considering the high concentration of Zn in the basal diet (84 mg Zn/kg DM). Perhaps, high dietary Zn concentrations replenished Zn storage before decreases in liver Zn were detected. While this has not been studied extensively, work by Niedermayer et al. [1] observed an increase in liver Zn concentrations 70 d post-administration of a high potency steroidal implant in steers. Data collected by Hufstedler and Greene [117] suggests this increase in liver Zn concentrations may be the result of upregulated Zn absorption due to steroidal implant administration. However, Dorton et al. [125] observed no liver Zn response to a moderate potency implant administered at the beginning of the growing and finishing phase in steers fed diets in excess of NASEM [46] Zn recommendations (45 to 51 mg Zn/kg DM). Therefore, liver Zn concentrations and absorption, may be more greatly affected with increasing steroidal implant potency.

These data indicate changes in plasma Zn concentrations may be more consistent than liver Zn concentrations due to steroidal implant administration. Although the detected differences in Zn concentrations remained within the wide reference ranges [60] for plasma (0.8–1.4 mg/mL) and liver (25 to 200 mg/kg DM), the corresponding performance differences suggest these changes are likely physiologically relevant. Interestingly, most of the observed changes in plasma Zn concentrations occurred within the aforementioned peak hormonal payout period of the steroidal implant (first 40 d post-implant administration) [19] and were associated with implant-induced improvements in growth [14,119,120]. Similarly, increased rate of gain in children recovering from malnourishment was correlated with lesser plasma Zn concentrations [126], again indicating circulating Zn is critical to support the growth response and may be an applicable biomarker of growth. Moreover, these Zn responses were more prominent in cattle receiving high potency steroidal implants. Indeed, these data indicate steroidal implants influence Zn metabolism. However, variables such as hormone potency, dietary Zn concentrations, and assessment of liver or plasma Zn concentration timing must be examined to further our understanding of steroidal implants’ effects on Zn metabolism and subsequent requirements of high growth rate cattle.

## 5. Conclusions and Future Directions

Strategic supplementation of TM, including Zn, to cattle based upon expected performance and management practices such as steroidal implant use is critical to a future that features precision cattle feeding. This review details the proposed multi-faceted interactions between Zn and steroidal implants on growth ranging from effects on hormone receptors to protein synthesis. The literature reveals supporting roles for additional dietary Zn in implanted cattle and many avenues for future research. With more than 20 million cattle on feed in the U.S. each year and Zn being a low-cost input, strategic supplementation of Zn could potentially better capture the growth performance advantages offered by steroidal implants while improving producer return on investment and the efficiencies of beef production. However, the optimal concentration of supplemental Zn to capitalize on the genetic potential and implant-induced growth rates of cattle is unclear. Similar to the energy and protein demands of implanted cattle, Zn requirements of the beef animal are likely increased to accommodate these higher rates of growth. An association between Zn metabolism and steroidal implant-induced growth, as outlined in this review, provide evidence of Zn as a potential limiting micronutrient in beef cattle growth.

Furthermore, optimal timing of Zn supplementation is unknown. Pinpointing a Zn supplementation window may prove useful in best accommodating the micronutrient demands of fed beef cattle. Phase feeding of greater concentrations of Zn during peak hormonal payout or other periods of high growth rates may optimize performance and best utilize TM resources. Supplementation of Zn at high concentrations in cattle poses minimal risk for toxicity, and supplemented concentrations are well below pharmacological concentrations fed in other species [49]. Therefore, plasma and liver Zn responses in implanted cattle reported in this review strongly suggest this Zn demand can be met within physiologically relevant rates of supplementation. In addition to determining the optimal concentration and timing of Zn supplementation for high growth animals, understanding how steroidal implants impact Zn absorption is vital to determining Zn supplementation strategies. To date, effects of steroidal implants on Zn absorption have only been evaluated in lambs. Replication of this work in cattle of both sexes utilizing varied potency of steroidal implants is necessary to ascertain how different steroidal implants influence absorption of Zn and other TM. Upon understanding the effects of steroidal implants on Zn absorption, Zn source should be further evaluated in implanted cattle to assess the use of more bioavailable Zn sources as a viable Zn supplementation strategy to accommodate implant-induced growth rates. Lastly, roles for Zn in growth processes and signaling is an area of continual discovery. Understanding how Zn influences hormone signaling and protein synthesis is critical to deepening our understanding of the many influential roles of Zn in steroidal implant-induced growth.

## Data Availability

Not applicable.
